# CRIMALDDI: a co-ordinated, rational, and integrated effort to set logical priorities in anti-malarial drug discovery initiatives

**DOI:** 10.1186/1475-2875-9-202

**Published:** 2010-07-13

**Authors:** Ian C Boulton, Solomon Nwaka, Ian Bathurst, Michael Lanzer, Donatella Taramelli, Henri Vial, Christian Doerig, Kelly Chibale, Steve A Ward

**Affiliations:** 1 TropMedPharma Consulting Ltd, 10 Brampton Chase, Lower Shiplake, Oxfordshire RG9 3BX, UK; 2 Special Programme for Research & Training in Tropical Diseases (TDR), World Health Organization, Avenue Appia 20, 1211 Geneva 27, Switzerland; 3 Medicines for Malaria Venture, PO Box 1826, ICC Building, 20, rte de Pré-Bois, 1215 Geneva 15, Switzerland; 4 Department of Parasitology, University of Heidelberg, 69120 Heidelberg, Germany; 5 Department of Sanità Pubblica-Microbiologia-Virologia, Università di Milano, Via Pascal 36, 20133 Milano, Italy; 6 Centre National de la Recherche Scientifique, UMR 5235, Université Montpellier 2, Place Eugene Bataillon, 34095 Montpellier Cedex 5, France; 7 Inserm-EPFL Joint Laboratory, Global Health Institute, Ecole Polytechnique Fédérale de Lausanne, CH-1015 Lausanne, Switzerland; 8 Department of Chemistry, University of Cape Town, Rondebosch, Cape Town, 7701, South Africa; 9 Liverpool School of Tropical Medicine, Pembroke Place, Liverpool L3 5QA, UK

## Abstract

Despite increasing efforts and support for anti-malarial drug R&D, globally anti-malarial drug discovery and development remains largely uncoordinated and fragmented. The current window of opportunity for large scale funding of R&D into malaria is likely to narrow in the coming decade due to a contraction in available resources caused by the current economic difficulties and new priorities (*e.g*. climate change). It is, therefore, essential that stakeholders are given well-articulated action plans and priorities to guide judgments on where resources can be best targeted.

The CRIMALDDI Consortium (a European Union funded initiative) has been set up to develop, through a process of stakeholder and expert consultations, such priorities and recommendations to address them. It is hoped that the recommendations will help to guide the priorities of the European anti-malarial research as well as the wider global discovery agenda in the coming decade.

## The Challenge

On October 17^th ^2007, Bill & Melinda Gates called on the global malaria community to embrace "an audacious goal--to reach a day when no human being has malaria and no mosquito on earth is carrying it" [1]. This call has re-energized the global commitment on malaria, building on previous initiatives, such as the WHO Roll Back Malaria Initiative. In Europe, the need to address communicable diseases linked to poverty has been recognized in several European Union Framework Programmes starting with FP5 in 2003, and subsequently FP6 and FP7. A strong focus on anti-malarial R&D was recognized at an early stage as a critical component of these programmes. The RBM Partnership's Global Malaria Action Plan also emphasized the need for an R&D agenda [2], which subsequently led to the launch of the MalERA Initiative [3]. MalERA is a process to identify R&D priorities that will be required to meet the needs of malaria elimination. For anti-malarial drugs, it attempts to redirect some of the R&D efforts from a short-term focus on malaria control agents towards drugs that will be more appropriate for elimination programmes [4].

Funding bodies consider many factors when deciding how to allocate their scarce resources. Important factors include (i) how the work will fit into a wider global research agenda, (ii) how this research agenda is responding to the needs of the disease endemic countries, and (iii) how their support is seen to be contributing in a positive and visible way to a broader global plan. The global agendas are often laid out in general and broad terms, sometimes lacking technical specificity and detail. They may also be too narrow in scope and fail to identify underlying obstacles (*e.g*. lack of suitable platform technologies) to progress in a particular field. Funders can benefit from detailed action plan proposals with input from all other stakeholders (endemic countries, researchers, policy makers, *etc*.) that allow them to set priorities with access to more information and context.

Agreed plans and strategies for anti-malarial drug R&D are essential communication tools for the community to interact with policy makers and funding bodies. There needs to be a constant dialogue, based on well-articulated arguments, in order to ensure that adequate resources continue to be made available and that they are targeted to bring maximum impact. Two rationales for maintaining support to drug R&D are the continuing need for new drugs and the need to remove obstacles to drug discovery.

## Continuous need for new drugs

One of the challenges for malarial drug R&D is to ensure sustained support even as the current efforts to control the disease start to show signs of success. In addition, better diagnosis will probably detect a drop of clinical cases, especially in sub-Saharan Africa. The new WHO recommendations to ensure that no treatment is given without a prior positive diagnosis through microscopy or RDTs will accelerate this trend [5,6]. The hope is that the positive outcome of a large reduction in cases will not lead policy makers to feel prematurely that the war against malaria is being won with the current tools and, therefore, resources can be redirected to other priorities. This was the mistake made for tuberculosis R&D with the roll-out of DOTS (Directly Observed Therapy - Short Course). The price that is now being paid is the scarcity of new drugs that are needed to meet the challenges of multidrug resistant and extensively drug resistant tuberculosis. The changing global environment for malaria control and elimination and its associated R&D must be constantly communicated to funding agencies and policy makers to ensure that discovery activities are not de-prioritized. This continuing need can be justified on several grounds:

### Resistance

The Plasmodium parasite continues to show a remarkable ability to develop resistance to new agents, despite efforts to find ways to minimize the risk [7]. The development and adoption of ACT (artemisinin-containing combination treatment) as first-line therapy for acute cases has been a major step forward in combating this problem [8]. It has also highlighted the potential utility of future new combinations that contain two or three complementary drugs with different modes of action (with or without an artemisinin component). Recent concerns in Cambodia about the loss of efficacy of artemisinins underlines the ability of the parasite to respond to new agents [9]. It also shows that the global malaria community cannot afford to assume that once it has a highly effective tool (such as ACT) this will reduce the need for new drugs with novel mechanisms of action to meet future (if not yet identified) resistance problems.

There is also the need to recognize that the problem of resistance is not only restricted to *Plasmodium falciparum*, as illustrated by the recent identification of evolving resistance of *Plasmodium vivax *to both chloroquine and primaquine [10]. There is a need to increase the priority of finding new drugs to tackle these problems if vivax as well as falciparum malaria is to be controlled and eliminated.

### Improving drug profiles

As malaria control and elimination programmes become more extensive and sophisticated, the profiles of the drugs needed for these programmes will change. As the community develops more integrated ways of delivering drugs treatment, the profiles of the drug combinations to be used in these systems have to adapt to the needs of these programmes. For example, the opportunities to deliver IPT (intermittent preventive treatment) alongside other interventions like childhood vaccination, school-age de-worming, maternal prenatal clinic visits, or bed net distributions, lead to an unmet need for new drug combinations that can be delivered in a single treatment (also possibly directly observed). Similarly, as the priority of treating the dormant liver stages of vivax malaria increases in malaria programmes outside sub-Saharan Africa, the profile of primaquine has been shown to be unsuitable and the need for a drug with a shorter dosage regimen increases [11]. The Medicines for Malaria Venture (MMV) and others are working to ensure that the malaria community has up-to-date and generally agreed target product profiles (TPPs) available to work towards [12,13].

### Changing objectives

Until quite recently, the focus of the global drive to control malaria has been on *Plasmodium falciparum*. This is understandable. Falciparum malaria is the major killer globally, notably of children and pregnant women, and it is the species that has given the most problems from the development of resistance. Now that global efforts against malaria are beginning to move beyond control to elimination, more emphasis has to be placed on *Plasmodium vivax *infections and developing agents that can be used against this species, especially in areas of mixed vivax and falciparum infections. Where elimination can be considered, then the removal of asymptomatic reservoirs of infection must also be considered. New agents that can be used in mass drug administration campaigns or other approaches are needed. All these changes in the objectives of delivering anti-malarial drug therapy underline the need for a continuing high level of investment in new drug R&D.

### Importance of other Plasmodium species

Another risk that must be recognized, as the control of *Plasmodium falciparum *and *Plasmodium vivax *improves, is the increase in importance of Plasmodium species that are currently seen as minor problems. The removal of falciparum and vivax may open up environmental gaps into which other species can enter. This will increase the need for drugs that are highly effective against other malarial species, for example *Plasmodium malariae, Plasmodium ovale*, and *Plasmodium knowlesi*. It is not currently clear if drugs with modes of action effective against vivax or falciparum will be adequately effective against these species once the competitive pressure of the major parasite species is removed.

## Increasing volumes of data

The pharmaceutical industry and other groups are releasing data on the structures of positive hits against Plasmodium derived from high throughput screening. This major initiative - facilitated by MMV - will give the malaria R&D community a vast number of structures to investigated. Two groups have already published large datasets of screening hits [14,15]. However, experience tells us that many of these structures will not be "druggable" as their activity against the parasite in an *in vitro *situation will not survive absorption or metabolism in a human host. Many structures will also be too closely related to currently identified molecules and so will probably not have the different modes of action to meet the target profiles required in the current era. Finally, there is a great risk that this large amount of data will not be properly prioritized and utilized. Too many groups may focus on a limited number of the most superficially promising structures and there will be unnecessary duplication of effort. This challenge will be exacerbated by a lack of transparency on who is working on which groups of molecules. The challenge will be to turn this volume of data into useful information about the molecules and who is working on them. This will help research groups and funders to make informed decisions on the most efficient way to use this information to design their research programmes and set their priorities.

## Obstacles to new drug discovery

Recently, attention is directed towards identifying molecules or modes of action that would be of potential value in the future fight against malaria. However, in comparison, there is less attention towards identifying potential methodological or platform technological obstacles to optimize the discovery process. For example, lack of screening methods (by parasite lifecycle stage or species), capacity in key elements of the discovery process (such as medicinal chemistry and predictive *in vivo *models), or understanding of the underlying biology, can all slow down or stall efficient drug discovery. These obstacles are not always given the priority they deserve when funding agencies make decisions on allocating resources. There is an understandable desire to get involved immediately in working with molecules and testing them against available models, rather than to develop better models or other less appealing, but necessary technologies or background scientific understanding. An additional roadblock to discovery is the concentration of relevant expertise and development organizations in the North with significantly less critical mass in disease endemic countries. Through structured co-operative initiatives it should be a possible to redress this imbalance.

## CRIMALDDI

The CRIMALDDI Consortium has been set up with a two-year EU Seventh Framework Programme grant [16]. Its objective is to help develop a framework that attempts to address the fragmented and often uncoordinated R&D initiatives in anti-malarial drug discovery and development. By bringing together experts (both from within the malaria world and outside) - through an integrated and logical series of meetings, conferences, and workshops - detailed recommendations on how to address some key priority challenges will be developed and then disseminated. Particular attention will be given to support leading European malaria research initiatives and partners to coordinate better their efforts. The Consortium aims to work in close collaboration with all relevant stakeholders (including funding bodies, academic research groups, the pharmaceutical industry, and policy makers both in malaria endemic and non-endemic countries). The Consortium will also aim to coordinate these efforts with international ones, attempt to engage the pharmaceutical industry, try to contribute towards the discussions on setting global research priorities, and give some input to the preparation of the European "anti-malarial drug" research agenda for the next decade. The work of the Consortium will focus on drug discovery and frameworks for translational research partnerships with organizations and endemic countries, as well as implication of these on drug development for sustained control and elimination. It is looking to make recommendations that will make a noticeable impact in the next decade. It is formulating its recommendations both around the continuing need for new drugs and the need to remove obstacles to drug discovery.

### Structure

The Consortium consists of nine participating institutions involved in anti-malarial drug discovery (see Table 1). The PIs representing each institutions form the Consortium Management Committee. The Management Committee is responsible for setting the workplan, identifying the priority challenges for in-depth analysis and recommendations, and overseeing the development of the outputs and proposals to the global community.

**Table 1:** CRIMALDDI Consortium Membership

Participating Institution	Principal Investigator
Liverpool School of Tropical Medicine	Prof Steve Ward
WHO/TDR	Dr Solomon Nwaka
Medicines for Malaria Venture	Dr Ian Bathurst
University of Heidelberg	Prof Michael Lanzer
University of Milan	Prof Donatella Taramelli
Centre National de la Recherche Scientifique (CNRS)	Prof Henri Vial
Inserm (Inserm-EPFL Joint Laboratory)	Prof Christian Doerig
University of Buea	Prof Simon Efange (also representing ANDI*)
University of Cape Town	Prof Kelly Chibale (also representing ANDI*)
	
**Consortium Co-ordinating Office**	
Mr Ian Boulton	Project Manager & Facilitator
Ms Susan Jones	EU Project Manager
Ms Tracy Seddon	Administrator

* ANDI is an initiative to promote & sustain African-led R&D innovation through the discovery, development, and delivery of affordable new tools including those based on traditional medicines [18].

The composition of the Consortium is drawn from European and African based institutions. In order to avoid taking too parochial view of the challenges confronting anti-malarial drug discovery or unduly focusing on their particular research priorities, the work of the Consortium is also subject to external review by an Expert Advisory Group (EAG). The EAG consists of key global opinion leaders in the field of anti-malarial drug discovery and development who are able to comment on the CRIMALDDI proposals both from the R&D and policy-makers' perspective. Its terms of reference are designed to ensure that the methodology and the recommendations of the Consortium are aligned with discussions by other similar agenda-setting initiatives and do not take too Eurocentric a view of the challenges. It enables the Consortium to co-ordinate its findings with other groups (especially MalERA). At its first meeting, the Management Committee of the Consortium identified the areas of expertise that it felt should be included in the EAG. It then identified appropriate experts to be invited who met these requirements and who would bring a wider geographical perspective to the Consortium's thinking. The EAG has met once and there are plans to meet twice in 2010.

### Methodology and work to-date

The basic approach of the project in arriving at its recommendations for the malaria community's consideration is to bring together global experts in a series of one- or two-day workshops. The starting point of each workshop is a detailed question that the participants can refine and then will use to develop interactively a set of detailed and action oriented recommendations designed to answer the question. One of the objectives of the Consortium is to try to bring new thinking from other fields of drug discovery to address the challenges that have been identified. It is hoped that the workshops will be able to find lessons from related fields (*e.g*. liver immunology in cancer, stem cell research, other infectious diseases) that may offer new insights worth pursuing in addressing the more intractable challenges found in malaria.

**Figure 1:**
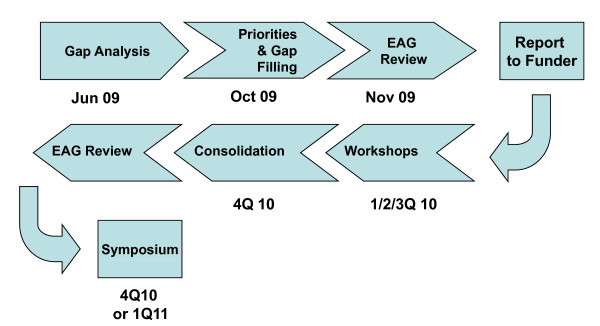
**CRIMALDDI Work Process**. The CRIMALDDI process is a logical and structured analysis of the challenges to novel anti-malarial drug R&D with the objective of delivering detailed action plans to address the priority challenges. The steps are (1) to identify the work needed to meet the GMAP objectives for drug treatment in both the Control & Elimination phases of the Plan: (2) identify the work currently being undertaken around the world and where there are gaps with the work needed: (3) prioritize the R&D efforts needed to fill the gaps, especially drug discovery and the development of platform technologies: (4) work with other experts to develop detailed action plans to fill the priority gaps: (5) consolidate the recommended action plans and publish them.

The overall process for the work of the Consortium is shown in Figure 1. The Consortium Management Committee has already met twice to identify the priority areas of focus for the Consortium (workstreams). The first meeting attempted to establish both the range of drug discovery and platform technology work required to meet the evolving needs for new anti-malarial drugs and the actual work being undertaken around the world. This then allowed the team to carry out a detailed gap analysis. At its second meeting, the team then grouped the gaps into logical workstreams, which could be possible topics for the focused workshops planned for 2010. The various workshop topics were then prioritized based on their importance to increasing the productivity of drug discovery and the degree of unmet need the Committee felt existed and the top five selected for further work (Table 2). Other topics considered are listed in Table 3. The rationale for addressing only the top five issues at this stage is presented. All this work was then subjected to review by the EAG before being taken further.

**Table 2:** Five priority workstreams

**Workstream No**.	Short Name	Workshop Question
1	*Pf *&* Pv *novel targets & classes	How to identify and exploit novel targets at all stages of the lifecycle of *P falciparum *&* P vivax*.
2	Managing the wealth of new HTS data	Given the large number of molecular structures that have given positive hits in the HTS screens and which are now being released, how to develop systems to:-
		Make the information available to the community in an accessible way;
		Filter the structures with robust methods to identify those structures which are druggable and more promising starts for lead optimization;
		Allow the community to know who is working on which structures so that duplication can be avoided and resources not unnecessarily wasted.
3	Artemisinin resistance	How to identify the mechanism(s) of artemisinin resistance in order to be able to design strategies to overcome or avoid it through novel combinations or novel molecular designs that counter the mechanism(s).
4	Stage-specific screening methods	How to develop a complete set of robust and replicable screening methods that can be used to screen novel compounds for efficacy against the various stages of the Plasmodium parasite lifecycle.
5	Using chemistry to understand biology	How to use the results of the whole cell screening of compounds for anti-malarial activity as a way of gaining insights into the underlying targets of different drug classes and then use this information to identify novel targets.

The five priority workstreams identified by the Consortium's analysis to-date and endorsed by the EAG.

**Table 3:** Workstreams Not Prioritized at this Stage

Workstream	Reason for not prioritizing at this stage
Novel combinations	Would first require the identification and development of new chemotypes from which to design appropriate novel combinations
Natural products	Considered an important route to identifying new drugs but would require broader discussion with experts specializing in this field than is possible in the first phase of the work of CRIMALDDI.
Novel drugs for severe malaria	Need appropriate models to study. Also likely that a first step would be to identify novel chemotypes and then compounds with the appropriate PK/PD properties for severe malaria.
Novel drugs for malaria in pregnancy	Need appropriate models to study. Also likely that a first step would be to identify novel chemotypes and then compounds with the appropriate PK/PD properties for malaria in pregnancy.
Drugs for mass administration	Starting point would be novel chemotypes from which drugs with appropriate efficacy & safety profiles can be identified.
Drugs to overcome resistance mechanisms (non-artemisinin)	The development of novel chemotypes would, by definition, identify molecular frameworks that could overcome resistance by different mechanisms. A specific effort to find molecules of the same class to overcome resistance would not be the most productive way forward.

These equally important workstreams were not included in this phase of the project for the reasons explained in the table. However, the Consortium or others may address them in the future.

The thinking of the Consortium members (endorsed by the EAG) in arriving at these five workshop topics can be summarized as follows:-

Workshop 1 ("*P. falciparum *and *P. vivax *novel targets and classes"): despite efforts to identify novel targets and chemotypes, much anti-malarial drug discovery is still focused on a few well-established and well-characterized groups (*e.g*. anti-folates, DHODH) and primarily on the blood stage of the parasite's life cycle. Are there ways to accelerate the identification of novel targets and drug classes that act elsewhere?

Workshop 2 ("Managing the wealth of new HTS data"): various research groups and pharmaceutical companies are placing data on large numbers of positive hits from whole cell malaria parasite high throughput screening into the public domain. However, there is a risk that there may not be adequate systems in place to allow this data to be turned by interested groups into meaningful information to support drug discovery. Are there one or more approaches to making this data available that will make it more useful and user-friendly to the wider malaria community?

Workshop 3 ("Artemisinin resistance"): since the identification of resistance to artemisinins in Cambodia, most of the research work on this topic has been focused on finding ways to prevent its spread outside the limited area where it is currently found. However, it is still unclear what the exact mechanism of this resistance is and the degree to which it may cause resistance to newer peroxide drug candidates. Is it possible to design an integrated research programme that will not only identify the mechanism of the resistance, but be able to be predictive of cross-resistance and also allow strategies to be developed to minimize its spread and preserve existing artemisinin-containing drugs?

Workshop 4 ("Stage-specific screening methods"): one of the barriers to anti-malarial drug discovery is the lack of robust screening methods that can be replicated by laboratories across the world. It is not possible to screen properly against all species of the human malaria parasite, or for activity against stages of the life cycle other than *Plasmodium falciparum *blood stage. Is it possible to develop recommendations on the priorities for research into new screens that are stage specific and can reliably identify species other than *P. falciparum*?

Workshop 5 ("Using chemistry to understand biology"): there is a large amount of information now available on the chemical structures that show activity against Plasmodium in whole cell screens. It ought to be possible to use information on the chemical structures that show activity to draw conclusions on the underlying parasite biology that is being targeted. What would a research programme to identify the biological processes that are being interrupted by the active chemotypes look like and what would need to be in place to support such a programme?

The next stage of the process will be to hold workshops to design detailed and action-oriented work plans to address the key issues identified. These workshops will be one or two days in duration. The Consortium will invite 10 to 20 global experts in the particular field of the question, but it will aim not to draw solely on experts from within the malaria community. Efforts will be made to bring in outside expertise that can bring new thinking on existing problems (*e.g*. what can be learned on issues of resistance from the anti-bacterial and anti-viral world). The selection of participants for the workshops is being made by the Workshop Leaders in consultation with the rest of the Management Committee and the EAG. They will attempt to identify global experts with the key skill sets and expertise to address in depth the topic of each Workshop. These workshops will be facilitated in order to ensure maximum interactivity and to keep the meeting focused on the specific question identified for analysis. The detailed questions to be addressed at the five priority workshops are shown in Table 2. If the workshops alone are not enough to develop adequate recommendations to the satisfaction of the participants, then consideration will be made to having additional meetings or other forms of collaborative discussions to enable quality answers to the workshop questions to be agreed upon. The outputs from the workshops will then be consolidated with the help of the EAG for publication at the end of the project. This will allow the recommendations to be available to the malaria community as one input to the agenda setting mentioned earlier in this paper.

The CRIMALDDI Consortium is aware of the need to align and coordinate its efforts with those of other related initiatives, most notably the Global Malaria Action Plan and MalERA. The presence of MMV and TDR as Consortium members is intended to ensure good coordination with existing efforts. The Chair of the Drug Discovery Workstream of MalERA is a member of the EAG. Members of the Consortium have also participated in meetings of the MalERA Drugs Consultative Group. As mentioned elsewhere, MalERA's focus is broad, high-level, and medium to long-term. CRIMALDDI's focus is on a few key priority areas where it plans to go in depth into their needs and where the impact can be seen in the next decade. Therefore the two initiatives are complementary.

### Community involvement and communications

The Consortium is well aware that it must try to gather input from as wide a range of stakeholders as possible. Formally it can rely on input from the members of the Consortium, the EAG, and the participants in the five workshops. They represent not only five European and two African leading drug discovery research centres in the Consortium. They also include key international organizations (TDR), funders (*e.g*. Bill and Melinda Gates Foundation, Wellcome Trust, MMV), major institutions (*e.g*. London School of Hygiene and Tropical Medicine, Institut Pasteur, Columbia University, Swiss Tropical and Public Health Institute), and the pharmaceutical industry. The geographical reach of the input covers not only European and African based institutions and organizations, but also ones based in India, South East Asia, and North America.

However the Management Committee decided at its first meeting that it needed to try to gather as wide range of input as possible from the anti-malarial community. To this end it has set up a website [17] where anyone will be able to view the outputs of the work to-date and to comment both on the outputs and on specific questions posed by the Consortium through the site's discussion forum. The members of the Consortium have and will continue to take every opportunity to raise the awareness of its work at relevant meetings (*e.g*. MIM Conference in Nairobi, November 2009).

The website will include detailed summaries of the work concluded to-date as well as the plans for the next steps. Minutes of the EAG will also be posted. In addition, the Consortium will publish summaries of the conclusion of the workstreams as they become available. Key issues that are raised at the workshops will be placed into the discussion section of the website so that non-participants in the workshops will have an opportunity to make their own comments. These will be taken into consideration when the final recommendations are written up. The Consortium plans to present its overall conclusions and recommendations at a major conference in late 2010 or early 2011.

## Conclusions

It is clear that the current window of opportunity of large scale resourcing for anti-malarial drug R&D will not last and that resources might become much more constrained over the next decade. It is vital to ensure that stakeholders worldwide are given clear and logical guidance on the priorities for such R&D. Research groups will then be able to take their cue from these priorities in deciding on the field of work they would like to engage in. It is hoped that this will ensure that the new anti-malarial drugs needed to meet the challenge of control, elimination, and eradication are developed in an efficient and logical manner. Long-term and broad ranging strategic plans (such as GMAP and MalERA) will be of great value in making clear the breadth of challenges to be addressed and identifying how the focus of the research agenda needs to be adapted over time. However, they will of necessity be high-level and may lack the technical detail that will help in establishing priority work plans needed to coordinate properly the R&D efforts in the coming decade. The CRIMALDDI Consortium is an initiative that is intended to contribute to the research agenda setting by making recommendations on a few key near-term priority needs so, it is hoped, anti-malarial R&D can proceed in the most efficient and effective way. It will develop recommendations for these priorities that may be then used to guide and coordinate the efforts, especially (but not exclusively) in Europe. Through the development of detailed and prioritized recommendations, it is hoped that the output of the Project will be a valuable tool for funding agencies and other stakeholders to set their priorities for drug discovery. The eventual realization of these priorities will require appropriate collaborative frameworks including participation of researchers from the global community incorporating malaria-endemic countries.

## List of abbreviations

ACT: artemisinin-containing combination treatment; ANDI: African Network for Drugs and Diagnostics Innovation; CRIMALDDI: The Coordination, Rationalization, and Integration of antiMALarial Drug Discovery & development Initiatives; DOTS: Directly Observed Therapy: Short course; EAG: Expert Advisory Group; EU: European Union; GMAP: Global Malaria Action Plan; IPT: Intermittent Presumptive Treatment; MalERA: Malaria Eradication Research Agenda; MIM: Multilateral Initiative on Malaria; MMV: Medicines for Malaria Venture; PI: Principal Investigator; RBM: Roll Back Malaria; RDT: Rapid Diagnostic Test; TDR: UNICEF/UNDP/World Bank/WHO Special Programme for Tropical Disease Research & Training; TPP: Target Product Profile.

## Competing interests

The authors declare that they have no competing interests.

## Authors' contributions

ICB and SAW prepared the first draft of paper. All authors contributed to the design of the project methodology and in preparing the paper. All authors have read and approved the final manuscript.
